# Socioeconomic Inequities in Adherence to Positive Airway Pressure Therapy in Population-Level Analysis

**DOI:** 10.3390/jcm9020442

**Published:** 2020-02-06

**Authors:** Abhishek Pandey, Suresh Mereddy, Daniel Combs, Safal Shetty, Salma I. Patel, Saif Mashaq, Azizi Seixas, Kerry Littlewood, Girardin Jean-Luis, Sairam Parthasarathy

**Affiliations:** 1 UAHS Center for Sleep & Circadian Sciences and Division of Pulmonary, Allergy, Critical Care and Sleep Medicine, Department of Medicine, University of Arizona, Tucson, AZ 85724, USA; apandey47@gmail.com (A.P.); s.mereddy76@gmail.com (S.M.); safalshetty@gmail.com (S.S.); salmapatel@deptofmed.arizona.edu (S.I.P.); saifmashaqi@deptofmed.arizona.edu (S.M.); 2 School of Social Work, College of Behavioral and Community Sciences, University of South Florida, Tampa, FL 33620, USA; littlewood@usf.edu; 3 UAHS Center for Sleep & Circadian Sciences and Division of Pulmonary and Sleep Medicine, Department of Pediatrics, University of Arizona, University of Arizona, Tucson, AZ 85724, USA; combs89@email.arizona.edu; 4 Department of Population Health NYU Langone, New York, NY 10016, USA; Azizi.Seixas@nyulangone.org (A.S.); Girardin.Jean-Louis@nyulangone.org (G.J.-L.)

**Keywords:** sleep apnea, adherence, positive airway pressure therapy, health disparities, big data, health policy, health equity

## Abstract

(a) Background: In patients with sleep apnea, poor adherence to positive airway pressure (PAP) therapy has been associated with mortality. Regional studies have suggested that lower socioeconomic status is associated with worse PAP adherence but population-level data is lacking. (b) Methods: De-identified data from a nationally representative database of PAP devices was geo-linked to sociodemographic information. (c) Results: In 170,641 patients, those in the lowest quartile of median household income had lower PAP adherence (4.1 + 2.6 hrs/night; 39.6% adherent by Medicare criteria) than those in neighborhoods with highest quartile median household income (4.5 + 2.5 hrs/night; 47% adherent by Medicare criteria; *p* < 0.0001). In multivariate regression, individuals in neighborhoods with the highest income quartile were more adherent to PAP therapy than those in the lowest income quartile after adjusting for various confounders (adjusted Odds Ratio (adjOR) 1.18; 95% confidence interval (CI) 1.14, 1.21; *p* < 0.0001). Over the past decade, PAP adherence improved over time (adjOR 1.96; 95%CI 1.94, 2.01), but health inequities in PAP adherence remained even after the Affordable Care Act was passed. (d) Conclusion: In a nationally representative population, disparities in PAP adherence persist despite Medicaid expansion. Interventions aimed at promoting health equity in sleep apnea need to be undertaken.

## 1. Introduction

Obstructive sleep apnea (OSA) is a prevalent condition that is most frequently treated with positive airway pressure (PAP) therapy [1,2]. However, non-adherence to PAP therapy has been noted in a high proportion of adults with OSA (46–83%) [3,4,5], and such poor adherence is associated with increased risk for fatal and non-fatal cardiovascular events [6,7]. Previously, investigators have suggested that adherence to PAP therapy may be worse in individuals belonging to a lower socioeconomic status (SES) [8,9,10,11]. Low SES and racial/ethnic minority populations have a greater burden of medical comorbidities associated with OSA, such as diabetes mellitus, hypertension, stroke, and higher cardiovascular mortality, which may place these groups at a particularly greater risk for OSA-related morbidity and mortality [12,13]. 

Specifically, Platt and colleagues showed that in the Veteran population in Philadelphia, PAP adherence (>4 h/night for 70% of nights) was great among higher SES neighborhoods (64%) compared to lower neighborhoods (34%) [8]. Similar findings were observed in the analysis of Canadian health administrative data where individuals living in higher income neighborhoods had a 27% greater chance of accepting PAP therapy when compared to the lowest income neighborhood [9]. Other studies have involved over five cities in the United States or participants at a single site in New Zealand, but, whether such regional findings are generalizable to population-level data in the United States is uncertain [10,11]. Geographic differences in practice trends [14], variable implementation of the health policy pertaining to PAP benefits [15,16,17], or even altitude with the effects on residual central apneas [18], could influence PAP adherence and patient outcomes [2]. We proposed to address such knowledge gaps by analyzing national population-level data from a PAP adherence database that spans 17 years (2000–2016).

PAP adherence has been proposed as a quality metric [19]. If PAP adherence is negatively influenced by a particular practice serving a disproportionately greater share of a health disparate (lower SES) population, then there are legitimate concerns that such practices could fail and consequently further accentuate or perpetuate health inequity [20]. A better understanding of health inequities pertaining to PAP adherence in patients with sleep apnea at a national-level would better prepare us to consider such data for adjustment of performance of practices with baseline differences in the proportion of individuals with socioeconomic disadvantage. A measure of such differences across practices in various states is operationalized by the Medicare Disproportionate Share Hospital (DSH) index [21]. It is pertinent to note here that the Affordable Care Act (ACA) reduced Medicare payments to hospitals that had a greater DSH index and such a policy could potentially negate the benefits of broadening health insurance coverage to the lower SES populations through Medicaid expansion [21]. We proposed to explore the effect of ACA over the 17 years of data that was available in our database.

## 2. Experimental Section

We performed a bioinformatics-based study whereby de-identified data obtained from the national-level adherence database (EncoreAnywhere^®^™, Philips-Respironics, Inc., Murrysville, PA, USA) was geo-linked to publicly available de-identified data that provides median household income information by 5-digit ZIP code of residence contained in both datasets [22,23]. Similarly, information regarding altitude was obtained and geo-linked because there is data suggesting that central apneas are worsened by high altitude and in a population-level analysis such apneas could influence adherence adversely [18,24]. Institutional Review Board approval was obtained from the University of Arizona (Protocol #1609849884). The study sponsor provided the data and funding but did not play a role in the conception, data-analysis, interpretation, or writing of the manuscript.

### 2.1. Adherence Data

De-identified data were extracted from the web-based patient management system that supports the monitoring of PAP therapy in patients with sleep apnea (EncoreAnywhere^®^, Philips-Respironics, Inc., Murrysville, PA, USA). Therapy data were uploaded from the patient’s PAP device to the database through wireless technology, Secure Digital (SD) cards, or telephone modem dating from 2000 to 2016. Database contained variables of interest including the following: (a) year of set-up; (b) 5-digit ZIP code (only 5 digits with no postal information smaller than a ZIP code); (c) device type; days of PAP therapy; (d) average daily adherence (hours and minutes); (e) average daily adherence (days used); (f) minimum usage hours; maximum usage hours; (g) percent days used; (h) percent days not used; (i) days with usage; (j) percentage of days with > 4 h of usage; (k) percentage of days with < 4 h of usage; (l) average leak; percentage of night spent in high leak; (m) apnea–hypopnea index; (n) central apnea index; (o) whether Medicare criteria for adherence was met; and (p) the days it took to meet such a Medicare adherence criterion. 

### 2.2. Socioeconomic Data and Confounders

Data on patient socioeconomic status as a proxy for individual socioeconomic status were obtained by linking publicly available de-identified data that provides median and mean household income information by respondent 5-digit ZIP code of residence with the United States Census [22]. Population denominator data and demographics were obtained from the U.S. Census collected at the ZIP Code Tabulation Area (ZCTA) level [23]. ZCTAs were created by the U.S. Census Bureau to follow census block boundaries and are similar to the ZIP Codes used by the U.S. Postal Service [25]. Adherence data were analyzed by the four quartiles of median income and adjusted for other potential confounders such as age, race, gender, and level of education. We were unable to determine the ZIP code location for some of the participants because information in the ZIP code field was unavailable. The final number of respondents in our analyses was 170,641 (63.7% of the available database).

### 2.3. Data-Analysis

Unadjusted comparison of various baseline characteristics across the four different quartiles of median household income groups was performed using Analysis of Variance (ANOVA), χ^2^ test, or nonparametric equivalent. We used univariate and then multivariate regression to evaluate if median income and year of set-up (PAP initiation) were associated with PAP adherence. We evaluated age, gender, race, ethnicity, device type, residual sleep-disordered breathing events (apnea–hypopnea index or central apnea index), and altitude of residence as covariates. Variables were included in the multivariate model if the *p*-value was < 0.10. We performed collinearity diagnostics and sensitivity analysis as appropriate. We analyzed group differences between various income groups and year of PAP set-up using Kaplan–Meier curves to visualize the time taken to achieve Medicare-defined adherence from the date of set-up; log rank test to compare the curves across various income groups or set-up years, and Cox proportional hazards regression to describe the effect of the determinants (various income groups) on time to Medicare-defined adherence. Statistical analyses were performed using the SPSS software (IBM SPSS, Version 24.0, IBM, Armonk, NY, USA). 

For our analyses of the effect of the Affordable Care Act on the time to Medicare-defined PAP adherence, we used multivariable regression, with a generalized linear model. The independent variable of interest was the interaction between timing after Medicaid expansion and median income quartile, which compared the average difference in time to time to Medicare-defined PAP adherence between the various income quartiles in the period before ACA expansion with that after expansion, with adjustment for covariates. Similar analysis was performed for 90 days and 120 days for effect of various income quartiles on time to Medicare-defined PAP adherence to determine the difference in differences between various income quartiles before and after the 90- or 120-day time point following PAP set-up date. 

## 3. Results

**Figure 1:**
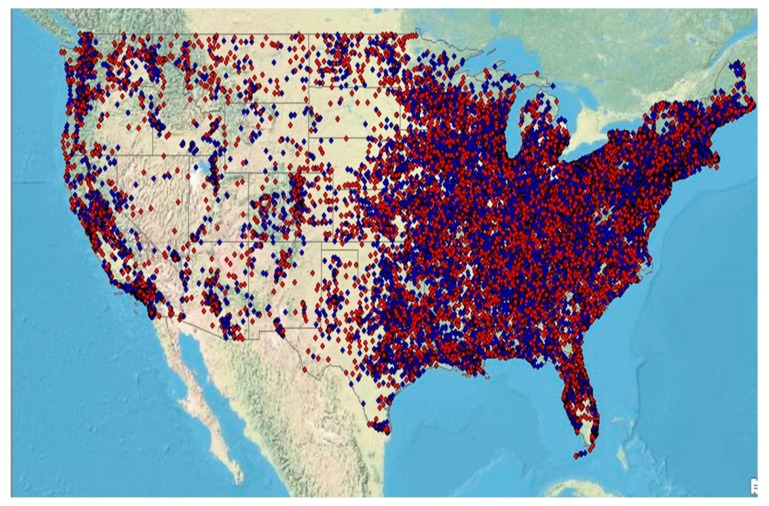
Geo-linked representation of individual patients who are adherent (blue symbols) or nonadherent to their positive airway pressure (PAP) therapy device. The data for 170,641 individuals (63.7%) of our available database with valid ZIP codes are shown. Note that more than half the symbols are red denoting a greater proportion of nonadherent individuals in this database.

We had complete data in 170,641 (63.7%) of the 267,793 individuals that were extracted from the database. The adherence data within the database contained the individual-level five-digit ZIP code data which was mapped to the U.S. Census collected at the ZIP Code Tabulation Areas (ZCTA) (Figure 1). 

Adherence data and demographic information are provided in Table 1. Sociodemographic variables were used to adjust for device adherence (outcome measure). In our dataset, the lowest income group (Quartile 1) had a median ZIP code household income less than $40,834 with progressively greater ZIP code-based household income in Quartile 2 ($40,834–50,366), Quartile 3 ($50,376–65,143), and Quartile 4 ($65,150–223,106). Demographics of participants by income quartile reveal differences in raw unadjusted characteristics across the four income groups (Table 1). Lower income groups had a greater proportion of individuals from racial/ethnic backgrounds (African Americans (Blacks), American Indians, and Hispanics) whereas higher income groups had a greater representation of Caucasians (Table 1). Automatic positive airway pressure (AutoPAP) devices were more likely to be prescribed to individuals in higher income groups than individuals in lower income groups (Table 1). 

**Figure 2:**
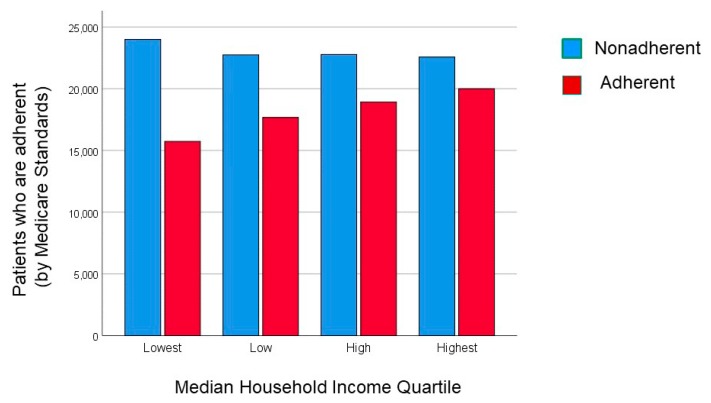
Number of patients who are adherent (red column) or nonadherent (blue columns) to positive airway pressure therapy by Medicare standards are shown by median household income quartile ranging from the lowest to the highest income levels. Note that the number of patients who are PAP adherent progressively increases as the median household income increases (χ^2^ < 0.0001). In our dataset, the lowest income group (Quartile 1) had a median ZIP code household income less than $40,834 with progressively greater ZIP code-based household income in Quartile 2 ($40,834–50,366), Quartile 3 ($50,376–65,143), and Quartile 4 ($65,150–223,106).

Raw unadjusted average daily adherence to PAP therapy was greater in higher income groups than lower income groups (Table 2; Figure 2). Proportion of days used as well as days when PAP device were used for more than 4 h per night was greater in the higher income group than the lower income group (Table 2). Adherence based upon Medicare rules requiring that PAP adherence be greater than 4 h per night for more than 70% of 30 consecutive nights in a 90-day period was also better in the higher income group when compared to the lower income group (Table 2). The proportion of time with high air-leak levels recorded by the PAP device was greater in the lower income than higher income group (Table 2). There were small statistically significant differences in residual apnea–hypopnea index (AHI) across the income groups, but these were not clinically significant differences and suggested that the prescribed setting of the PAP devices was effective in treating the sleep-disordered breathing that they were intended to treat. 

Raw unadjusted adherence by device type revealed best adherence for autoPAP devices followed by progressively lower levels of adherence to continuous positive airway pressure (CPAP), bilevel PAP, auto-bilevel PAP, adaptive servo-ventilation (ASV), and averaged volume assured pressure support (AVAPS; Table 3; ANOVA *p* < 0.0001). 

Univariate regression of various determinants of PAP adherence is provided in Table 4. Multivariate regression analysis revealed that independent of various confounders and group differences, adherence to PAP device was worse in lower income groups (Table 5). Younger age, men, African American race, and Hispanic ethnicity were independently associated with worse adherence. Elevated leak levels (average leak > 45 L per minute) was associated with worse adherence to PAP therapy. Elevated residual AHI and residual central apnea index were associated with worse PAP adherence (Table 5). Unlike the unadjusted PAP adherence data that revealed better adherence with autoPAP devices, after adjusting for confounders including household income (considering that autoPAP devices were more likely to be prescribed in higher income groups), CPAP devices had better adherence than all other PAP devices (Table 5). Greater altitude was progressively and independently associated with better adherence (Table 5).

**Figure 3:**
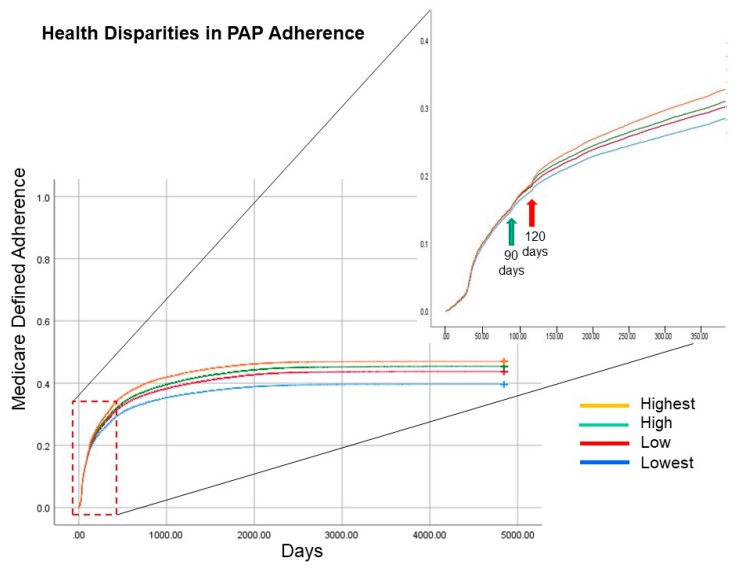
Kaplan–Meir curves of time to achieve Medicare-defined adherence to positive airway pressure (PAP) therapy device are shown for various income quartiles. Note that individuals from higher income neighborhoods are more likely to become adherent by Medicare-defined criteria sooner in time and also a greater proportion of individuals from a higher income neighborhood are likely to be adherent to PAP therapy (Log Rank test, *p* < 0.0001). The inset reveals a distinct pattern of emergent differences at the 90-day and again at the 120-day timepoints suggesting an effect of the 90-day Medicare rule that threatens to discontinue benefits in individuals who are nonadherent at that point in time (difference-in-difference analysis; *p* < 0.0001).

Temporal patterns of adherence for individual patients as well as for the entire cohort over the past 17 years for all patients were evaluated. For the former, the time taken to achieve adherence based upon Medicare definition was greater in the lower income groups than higher income groups (Figure 3; Log Rank; *p* < 0.0001). A closer review of the time point when differences between the income quartile groups began points to the inflection at the 90 and 120-day time points (Figure 3 inset). The difference-in-difference analysis performed as time and income group interaction term revealed that—when compared to before 90-days—after 90-day timepoint revealed a greater difference in time taken to achieve Medicare-defined adherence across all four income groups (General Linear Model; *p* < 0.0001 for all four income groups). Similarly, the difference-in-difference analysis for the 120-day timepoint revealed a greater difference in time taken to achieve Medicare-defined adherence across all four income groups (General Linear Model; *p* < 0.0001 for all four income groups).

**Figure 4:**
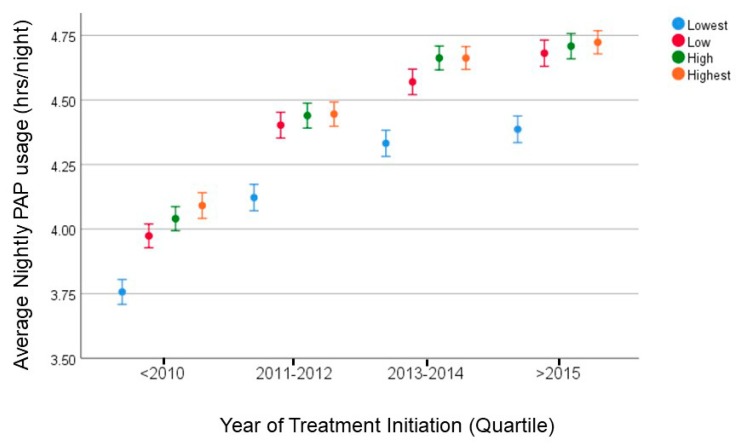
Raw unadjusted adherence to positive airway pressure (PAP) device (with 95% confidence intervals) is shown for various income quartiles as a function of year of set-up or initiation of the PAP device. Notice that there is a clear trend for improvement in adherence as a function of time, but disappointingly the health inequities remain even after 2014 when the Affordable Care Act and Medicaid expansion occurred.

**Figure 5:**
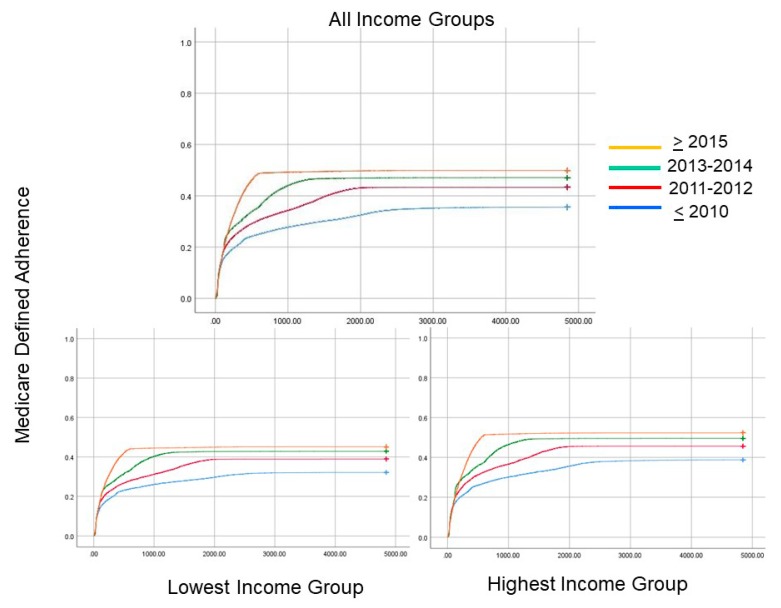
**(a)** The upper panel shows Kaplan–Meir curves that reveal differences in the temporal pattern of adherence to positive airway pressure (PAP) therapy (by Medicare-defined criteria) based upon the year of set-up or PAP initiation. Note that progressively after 2010 there is a greater proportion of individuals who are adherent by Medicare standards and they accomplish such adherence earlier in time after their device is set-up (Log Rank; *p* < 0.0001). The lower panels reveal similar graphs for the (**b**) lowest and (**c**) highest income groups.

For the temporal pattern over the past 17 years, with various time periods expressed as quartiles by year for PAP device set-up, progressively more recent calendar year of PAP therapy initiation was associated with greater PAP adherence (Table 4, Table 5 and Table 6), but the health inequities in adherence across the income groups persisted (Figure 4). Kaplan–Meier plots of adherence by income groups revealed an effect of the year of set-up on time to PAP adherence by Medicare criteria occurring sooner as a function of time since PAP set-up (Figure 5; Log Rank; *p* < 0.0001). However, difference-in-difference analysis across the various income groups did not reveal any effect of the 2014 ACA on health inequities in PAP adherence when compared to before 2014 (*p* = 0.7).

## 4. Discussion

We found health inequities in a nation-wide analysis of PAP adherence. Such findings are consistent with other prior reports of health inequities from state or multiple city datasets [8,10,11]. Similar to prior findings, PAP adherence was worse in certain groups of individuals who are overrepresented in lower income households such as African Americans, Native Americans, and individuals of Hispanic ethnicity. The association between poor PAP adherence and individuals with lower median household income was robust and persisted after adjusting for other known confounders such as age, sex, race-ethnicity, device type, and even altitude. We are uncertain as to the basis for the relationship between better adherence and greater altitude, as there are no prior reports of such findings to our knowledge. Conceivably, the greater degree of nocturnal hypoxia with higher altitude could render the sleep apnea to engender nocturnal dyspnea and greater daytime symptoms which may, in turn, promote better adherence [26].

We found two time-based effects that pertain to patient-level data as well as temporal change in adherence over the 17-year time period by the year of PAP device initiation or set-up. First, for the patient-level temporal pattern, we noticed that the inequities in adherence began at the 90-day period which corresponds to the “90-day Medicare rule”. Essentially, the Center for Medicare and Medicaid Services (CMS) propounded a National Carrier Determination for PAP benefits in 2008 which proposed a 12-week trial period (90 days) soon after PAP device set-up. This 90-day rule proposes that for continued PAP benefits, the CMS beneficiary needs to use the PAP device in an adherent manner (at least 70% of nights > 4 h per night over a 30 consecutive day period within the 90-day trial period) and benefit symptomatically. Failing the achievement of PAP adherence, depending on the insurance type, individuals may lose PAP medical benefits or may need to make a visit to see a provider in order to maintain continued insurance coverage for the PAP therapy. The Kaplan–Meir plots were revealing in that they showed separation or appearance of differences in Medicare-defined PAP adherence at the 90- and 120-day time points (Figure 3 inset). Difference-in-difference analysis suggested that both the 90-day and 120-day time points were associated with the separation of the Kaplan–Meir curves across the four income groups. Anecdotally, through stakeholder engagement of home care companies we understand that nonadherent individuals may have an additional 30-day period before the device is actually retrieved and that soon after the 90th day is when they begin receiving telephone calls or written correspondence requesting the retrieval of the machine or noncoverage of PAP benefits. There are other factors that may play a role in the appearance of such inequities in the 90 to 120-day time period. For example, during this time period is when the availability of more mask choices may be required to achieve adequate mask seal and comfort. The lack of an adequate mask seal may lead to higher levels of leak that may, in turn, lead to nonadherence [27]. Such a theory is supported by our finding that mask leak levels were indeed greater in the lower income groups than the higher income groups (Table 4 and Table 5). The ability of individuals from a higher income group to purchase PAP accessories (mask, mask liners, head gear) out-of-pocket when compared to those in lower income groups or greater health literacy levels leading to greater likelihood for PAP adherence when faced with the threat of losing the therapy device cannot be discounted [28,29,30,31,32]. Conversely, reducing out-of-pocket costs can lead to better adherence to medications in many disease conditions [30]. In line with such reasoning, we found that the adherence in the top three quartiles of income groups to be closer with a steep fall-off in adherence in the lowest income quartile (Figure 4). Other factors such as access to care in accredited centers and sleep-certified providers may also potentially play a role, but this is speculative on our part [33]. 

A second time-based effect was the association between the year of set-up and adherence (Figure 4 and Figure 5; Table 5). There is a clear temporal effect with improved adherence over the past 16 years. However, it is worrisome that the health inequities in PAP adherence persisted over this time period without signs of narrowing. Such persistence speaks to the need for effective interventions aimed at improving health equity [34]. However, we know that effecting health equity in general requires interventions at multiple levels (patient and health policy level). The expansion of Medicaid in most states in 2014 can improve patient outcomes, but in our analysis, there was no change in health inequities after 2014 in our national-level database [35]. Such a finding was not unexpected considering that the Affordable Care Act did not address the 90-day policy for PAP coverage which was enacted in 2008 [36]. It is interesting that there has been a progressive improvement in Medicare-defined adherence being achieved earlier over the past 17 years (Figure 4). It is uncertain as to whether such a trend is attributable to the 90-day rule or improvements in device and mask interface technology. 

The device technology surprisingly revealed greater adherence to CPAP when compared to other PAP devices after adjusting for household income. CPAP devices had better adherence than all other PAP devices after adjusting for confounders including household income (Table 5) Although in univariate regression, the autoPAP device was associated with greater adherence (Table 4), however, considering that autoPAP devices were more likely to be prescribed in higher income groups (Table 1), in multivariate regression, autoPAP devices was not associated with worse adherence when compared to CPAP. Such findings suggest that the more expensive autoPAP devices were likely associated with better adherence through their association with higher median household income. The findings of worse adherence in younger when compared to older individuals, men when compared to women, are notably small in effect size despite a large sample size and are consistent with prior reports suggesting a lack of a consistent association [3]. The findings of poor CPAP adherence in the African American race and Hispanic ethnicity are similar to prior reports [3].

An important consideration that needs discussion is the clinical significance of the effect size of the association between socioeconomic status (measured as median household income) and PAP adherence in our study. Most ascertainments of clinically meaningful differences in adherence are derived from clinical trials or consensus of experts rather than real-world population-based studies. Although, the average adherence measured as hours per day is seemingly small between the extremes of median household income (namely, 0.4 h per day or 24 min per day) the proportion of individuals who are nonadherent as per Medicare criteria is much more significant at 7.4% (Table 2; approximately 2,940 patients in the lowest income quartile). This is because a small difference in usage per night (of 0.4 h per night) that occurs when a population suffers from borderline levels of adherence close to 4 h per night (4.1 h per day for the lowest median income group (Table 2)) can have a much larger consequence to their Medicare adherence status when compared to a seemingly small reduction in usage per day. Essentially, the clinically meaningful difference in PAP adherence may become artifactually smaller when a health policy rule arbitrarily derives a threshold level of adherence that is very close to the average adherence level of a population and on which continued PAP therapy medical benefits are based upon. In our population study, essentially 2940 (7.4%) of 39,727 patients could lose their PAP devices. Such an observation should make us rethink such arbitrary thresholds for PAP adherence when considering “real-world” populations.

## 5. Conclusions

In conclusion, in a nationally representative population of patients with sleep apnea, socioeconomic inequities in PAP adherence persist despite Medicaid expansion. Considerations for change in health policy for individuals in lower income neighborhoods in addition to patient-level interventions aimed at promoting PAP adherence would be responsive to calls for promoting health equity in lower income populations [5,29,37,38,39]. Moreover, we should exercise caution in implementing PAP adherence as a quality metric for healthcare practices or alternatively consider adjusting for the DSH index if PAP adherence were adopted as a quality metric. Moreover, more could be accomplished to reduce health inequities in patients with sleep apnea by increasing DSH payments to offset a growing number of insured low-income population that was appropriately facilitated by the ACA.

## Figures and Tables

**Table 1:** Demographics of participants by income quartile.

	Quartile 1	Quartile 2	Quartile 3	Quartile 4	*p*-Value
	−39,727	−40,406	−41,691	−42,559	
Age	37.8 ± 6.2	38.9 ± 5.8	40 ± 5.3	39.7 ± 4.6	<0.0001
Age groups					<0.0001
≤19	9585 (5.8%)	10,326 (6.3%)	11,293 (6.9%)	13,051 (7.9%)	
20–39	9830 (6.0%)	10,712 (6.5%)	11,810 (7.2%)	11,941 (7.3%)	
40–59	8832 (5.4%)	10,295 (6.3%)	11,896 (7.2%)	14,492 (8.8%)	
60–79	5071 (3.1%)	5885 (3.6%)	6458 (3.9%)	6859 (4.2%)	
>80	1291 (0.8%)	1577 (1.0%)	1613 (1.0%)	1629 (1.0%)	
Sex (Male)	9955 (48.6%)	11,205 (48.8%)	12,425 (48.7%)	13,846 (48.7%)	<0.05
Race					<0.0001
Caucasian	25,285 (63.6%)	31,126 (77%)	39,059 (78.6%)	34,116 (80.2%)	
Black	10,499 (26.4%)	5330 (13.2%)	5671 (11.4%)	3536 (8.3%)	
American Indian					
Asian	345 (0.9%)	311 (0.8%)	1990 (0.6%)	179 (0.4%)	
Hawaiian	853 (2.1%)	1142 (2.8%)	1990 (4%)	3247 (7.6%)	
Other	43 (0.1%)	64 (0.2%)	111 (0.2%)	104 (0.2%)	
	2703 (6.8%)	2433 (6.0%)	2545 (5.1%)	1377 (3.2%)	
Ethnicity					<0.0001
Hispanic	3211 (15.7%)	3167 (13.8%)	3189 (12.5%)	2599 (9.2%)	
Not Hispanic	17,251 (84.3%)	19,806 (86.2%)	22,316 (87.5%)	25,802 (90.8%)	
Elevation	596 (272, 940)	648 (290, 953)	529 (110, 814)	391 (58, 709)	<0.0001
Year of set-up					<0.0001
<2010	10,481 (26.4%)	11,039 (27.3%)	10,740 (25.8%)	9284 (21.8%)	
2011–2012	9644 (24.3%)	9860 (24.4%)	10,309 (24.7%)	10,372 (24.4%)	
2013–2014	9977 (25.1%)	10,015 (24.8%)	10,809 (25.9%)	11,642 (27.4%)	
>2014	9625 (24.2%)	9492 (23.5%)	9833 (23.6%)	11,261 (26.5%)	
Device type					<0.0001
CPAP	15,633 (39.4%)	14,375 (35.6%)	14,925 (35.8%)	14,995 (35.2%)	
Bilevel PAP	2434 (6.1%)	2318 (5.7%)	2114 (5.1%)	2127 (5.0%)	
AutoPAP	17,206 (43.3%)	18,981 (47.0%)	20,308 (48.7%)	21,396 (50.3%)	
Auto-bilevel	3822 (9.6%)	4013 (9.9%)	3513 (8.4%)	3231 (7.6%)	
ASV	548 (1.4%)	653 (1.6%)	724 (1.7%)	674 (1.6%)	
AVAPS	84 (0.2%)	66 (0.2%)	107 (0.3%)	136 (0.3%)	

CPAP = continuous positive airway pressure; PAP = positive airway pressure; ASV = adaptive servo-ventilation; AVAPS = averaged volume assured pressure support.

**Table 2:** Device adherence information.

	Quartile 1	Quartile 2	Quartile 3	Quartile 4	*p*-Value
	−39,727	−40,406	−41,691	−42,559	
Average daily adherence (all days) (hours/day)	4.14 ± 2.57	4.39 ± 2.52	4.46 ± 2.49	4.50 ± 2.44	<0.0001
Average daily adherence (days used) (hours/day)	5.39 ± 2.21	5.59 ± 2.13	5.63 ± 2.08	5.64 ± 2.04	<0.0001
Percent days used (%)	71.5 ± 28.9	73.8 ± 28.0	74.8 ± 27.5	75.3 ± 27.0	<0.0001
Percent days used with >4 h nightly use (%)	53.4 ± 33.6	57.1 ± 33.0	58.4 ± 32.8	59.5 ± 32.5	<0.0001
CMS adherence, n (%)	15,736 (39.6%)	17,671 (43.7%)	18,925 (45.4%)	19,996 (47%)	<0.0001
Average leak (L/min)	32.5 ± 23.4	32.3 ± 22.1	31.4 ± 21.1	31.0 ± 21.0	<0.0001
Percent of night with high leak	5.9 ± 12.5	5.4 ± 12.9	4.8 ± 11.1	4.3 ± 10.6	<0.0001
Median (IQR)	0.8 (0.1, 5.2)	0.7 (0.1, 4.3)	0.6 (0.0, 3.5)	0.5 (0, 3.0)	<0.0001
Residual Apnea Hyponea–Index	3.9 ± 5.3	4.0 ± 5.1	3.9 ± 5.1	4.0 ± 5.2	0.004
Median (IQR); (events/hour)	2.4 (1.1, 4.7)	2.5 (1.2, 4.8)	2.5 (1.2, 4.8)	2.5 (1.2, 4.9)	<0.0001
Residual Central Apnea Index	0.6 ± 1.8	0.7 ± 1.8	0.7 ± 1.9	0.7 ± 1.9	<0.0001
Median (IQR) (events/hour)	0.1 (0, 0.5)	0.2 (0, 0.6)	0.2 (0, 0.6)	0.2 (0, 0.6)	<0.0001

CMS = Center for Medicare Medicaid Services (Medicare); IQR = interquartile range; L/min = liters per minute.

**Table 3:** Adherence by device type *.

Device Type	Adherence	Proportion of Days with > 4 h per Night
CPAP	4.38 ± 2.5	57.3 ± 33.0
Bilevel PAP	4.20 ± 2.66	53.78 ± 34.11
AutoPAP	4.44 ± 2.47	58.26 ± 32.72
AutoBilevel PAP	4.20 ± 2.63	53.48 ± 33.77
ASV	4.00 ± 2.48	51.22 ± 32.29
AVAPS	3.10 ± 2.58	37.70 ± 31.52

CPAP = continuous positive airway pressure; PAP = positive airway pressure; ASV = adaptive servo-ventilation; AVAPS = averaged volume assured pressure support. ANOVA *p*-value < 0.0001; All post-hoc comparisons were different from each other except for bilevel PAP and auto-bilevel PAP. * Hours of usage per night.

**Table 4:** Univariate regression of adherence to PAP therapy.

Device Type	Adjusted OR (95% CI)	*p*-Value
Median Income *		*p* < 0.0001
Low ($40,834–50,366)	1.19 (1.15, 1.22)	
High ($50,376–65,143)	1.27 (1.23, 1.30)	
Highest ($65,150–223,106)	1.35 (1.31, 1.39)	
Age	1.01 (1.01, 1.01)	*p* < 0.0001
Women	1.00 (1.00, 1.00)	*p* < 0.0001
African American race	0.99 (0.99, 0.99)	*p* < 0.0001
Hispanic ethnicity	0.99 (0.99, 0.99)	*p* < 0.0001
Average leak > 45 lpm	0.76 (0.75, 0.78)	*p* < 0.0001
Device type ^¶^		*p* < 0.0001
Bilevel PAP	0.87 (0.83, 0.91)	
AutoPAP	1.05 (1.03, 1.07)	
AutoBilevelPAP	0.83 (0.79, 0.86)	
ASV	0.68 (0.63, 0.74)	
AVAPS	0.30 (0.23, 0.38)	
High altitude elevation ^†^		*p* < 0.0001
2000–4000 feet	1.06 (1.01, 1.12)	
4001–6000 feet	1.24 (1.16, 1.32)	
>6000 feet	1.15 (1.02, 1.31)	
Residual CAI > 5/hour	0.59 (0.55, 0.63)	*p* < 0.0001
Residual AHI > 5/hour	0.62 (0.61, 0.64)	*p* < 0.0001
Set-up year ^‡^		*p* < 0.0001
Quartile 2 (2011–2012)	1.39 (1.35, 1.42)	
Quartile 3 (2013–2014)	1.61 (1.57, 1.66)	
Quartile 4 (>2015)	1.80 (1.75, 1.85)	

OR = odds ratio; 95%CI = 95% confidence interval; * reference is Quartile 1 (lowest income quartile; <$40,834); ANOVA *p*-value < 0.0001; lpm = liters per minute; **^¶^** referenced against CPAP; automatic devices (autoPAP, auto-bilevel PAP, adaptive servo-ventilation (ASV), adaptive volume averaged pressure support (AVAPS)). † High elevation (referenced against < 2000 feet); CAI = central apnea index; AHI = apnea–hypopnea index. **^‡^** Reference earliest quartile of time (in years) of set-up is < 2011.

**Table 5:** Multivariate logistic regression of adherence to PAP therapy.

Device Type	Adjusted OR (95% CI)	*p*-Value
Median Income *		*p* <0.0001
Low ($40,834–50,366)	1.12 (1.08, 1.15)	
High ($50,376–65,143)	1.17 (1.14, 1.21)	
Highest ($65,150–223,106)	1.18 (1.14, 1.21)	
Age	1.01 (1.01, 1.01)	*p* < 0.0001
Women	1.01 (1.01, 1.01)	*p* < 0.0001
African American race	0.99 (0.99, 0.99)	*p* < 0.0001
Hispanic ethnicity	0.99 (0.99, 0.99)	*p* < 0.0001
Average leak > 45 lpm	0.78 (0.76, 0.79)	*p* < 0.0001
Device type ^¶^		*p* < 0.0001
Bilevel PAP	0.89 (0.84, 0.92)	
AutoPAP	0.92 (0.89, 0.94)	
AutoBilevelPAP	0.86 (0.83, 0.89)	
ASV	0.64 (0.59, 0.70)	
AVAPS	0.28 (0.22, 0.36)	
High altitude elevation (quartile) ^†^	1.14 (1.07, 1.21)	*p* < 0.0001
Residual CAI > 5/hour	0.62 (0.57, 0.66)	*p* < 0.0001
Residual AHI > 5/hour ^‡^	0.63 (0.62, 0.65)	*p* < 0.0001
Set-up year °		*p* < 0.0001
Quartile 2 (2011–2012)	1.44 (1.40, 1.48)	
Quartile 3 (2013–2014)	1.67 (1.63, 1.72)	
Quartile 4 (>2015)	1.96 (1.90, 2.01)	

OR = odds ratio; 95%CI = 95% confidence interval; * reference is Quartile 1 (lowest income quartile; <$40,834); ANOVA *p*-value < 0.0001; lpm = liters per minute; **^¶^** referenced against CPAP; automatic devices (autoPAP, auto-bilevel PAP, adaptive servo-ventilation (ASV), adaptive volume averaged pressure support (AVAPS)); † High elevation (step increase in quartile of elevation); CAI = Central Apnea Index; AHI = apnea–hypopnea index. **^‡^** Considering that AHI was collinear to CAI, this regression was done separately in lieu of CAI. ° Reference year of set-up category is < 2011.

**Table 6:** Cox proportional hazards for adherence to PAP therapy.

Device Type	Adjusted HR (95% CI)	*p*-Value
Median Income *		*p* < 0.0001
Low ($40,834–50,366)	1.08 (1.06, 1.10)	
High ($50,376–65,143)	1.11 (1.09, 1.14)	
Highest ($65,150–223,106)	1.12 (1.10, 1.15)	
Age	1.01 (1.01, 1.01)	*p* < 0.0001
Women	1.01 (1.01, 1.01)	*p* < 0.0001
African American race	0.99 (0.99, 0.99)	*p* < 0.0001
Hispanic ethnicity	0.99 (0.99, 0.99)	*p* < 0.0001
Average leak > 45 lpm	0.86 (0.84, 0.88)	*p* < 0.0001
Device type ^¶^		*p* < 0.0001
Bilevel PAP	0.93 (0.90, 0.96)	
AutoPAP	0.94 (0.92, 0.95)	
AutoBilevelPAP	0.90 (0.88, 0.93)	
ASV	0.76 (0.71, 0.81)	
AVAPS	0.36 (0.29, 0.45)	
High altitude elevation (quartile) ^†^	1.06 (1.05, 1.08)	*p* < 0.0001
Residual CAI > 5/hour	0.71 (0.67, 0.76)	*p* < 0.0001
Residual AHI > 5/hour ^‡^	0.73 (0.72, 0.75)	*p* < 0.0001
Set-up year °		*p* < 0.0001
Quartile 2 (2011–2012)	1.32 (1.29, 1.34)	
Quartile 3 (2013–2014)	1.50 (1.46, 1.53)	
Quartile 4 (>2015)	1.72 (1.70, 1.76)	

HR = hazard ratio; 95%CI = 95% confidence interval; * reference is Quartile 1 (lowest income quartile; <$40,834); lpm = liters per minute; **^¶^** referenced against CPAP; automatic devices (autoPAP, auto-bilevel PAP, adaptive servo-ventilation (ASV), adaptive volume averaged pressure support (AVAPS)); † high elevation (step increase in in quartile of elevation); CAI = central apnea index; AHI = apnea–hypopnea index. **^‡^** Considering that AHI was collinear to CAI, this regression was done separately in lieu of CAI. ° Reference year of set-up category is before the year 2011.
